# Discovering novel phenotypes with automatically inferred dynamic models: a partial melanocyte conversion in *Xenopus*

**DOI:** 10.1038/srep41339

**Published:** 2017-01-27

**Authors:** Daniel Lobo, Maria Lobikin, Michael Levin

**Affiliations:** 1 Department of Biological Sciences, University of Maryland, Baltimore County, 1000 Hilltop Circle, Baltimore, MD 21250, USA; 2 Allen Discovery Center at Tufts University, and Department of Biology, Tufts University, 200 Boston Avenue, Suite 4600, Medford, MA 02155, USA

## Abstract

Progress in regenerative medicine requires reverse-engineering cellular control networks to infer perturbations with desired systems-level outcomes. Such dynamic models allow phenotypic predictions for novel perturbations to be rapidly assessed *in silico*. Here, we analyzed a *Xenopus* model of conversion of melanocytes to a metastatic-like phenotype only previously observed in an all-or-none manner. Prior *in vivo* genetic and pharmacological experiments showed that individual animals either fully convert or remain normal, at some characteristic frequency after a given perturbation. We developed a Machine Learning method which inferred a model explaining this complex, stochastic all-or-none dataset. We then used this model to ask how a new phenotype could be generated: animals in which only some of the melanocytes converted. Systematically performing *in silico* perturbations, the model predicted that a combination of altanserin (5HTR2 inhibitor), reserpine (VMAT inhibitor), and VP16-XlCreb1 (constitutively active CREB) would break the all-or-none concordance. Remarkably, applying the predicted combination of three reagents *in vivo* revealed precisely the expected novel outcome, resulting in partial conversion of melanocytes within individuals. This work demonstrates the capability of automated analysis of dynamic models of signaling networks to discover novel phenotypes and predictively identify specific manipulations that can reach them.

One of the key areas in which artificial intelligence and the information sciences can contribute to biology is by helping human scientists understand cellular behavior in the context of a complex organism^1^,^2^. Computational methods can reverse-engineer mechanistic models of tissue patterning and shape formation from expression data and experimental phenotypes^3^,^4^,^5^,^6^,^7^,^8^,^9^. The utility of these methods is their ability to find novel regulatory interactions^10^ and even novel necessary regulatory genes^11^. These methods are indeed becoming indispensable for understanding the complex coordination of signals necessary to develop and maintain correct body shapes and organs. Moreover, such methods are required in order to develop interventions to make rational changes to complex anatomy and physiology, in the context of regenerative medicine and systems-level diseases such as cancer^12^.

The coordination of cellular behavior towards the anatomical needs of the host organism, and away from tumorigenesis, is achieved in part via bioelectrical communication among many cell types^13^,^14^,^15^,^16^,^17^,^18^,^19^. Recent work showed that depolarization of resting potential in a special cell population in *Xenopus* embryos, so-called instructor cells, results in a metastatic-like conversion of normal melanocytes^20^. Throughout the body (including at considerable distance from the depolarized region), these pigment cells become hyper-proliferative, acquire a highly arborized morphology, and invade organs and blood vessels, colonizing ectopic regions of the body. This conversion recapitulates the main features of a melanoma-like phenotype (including up-regulation of cancer-related genes)^21^. It is quite distinct from pigment changes occurring due to light exposure^22^,^23^,^24^ because it affects not the pigment level of individual melanocytes but instead triggers drastic invasiveness that does not significantly change as a function of light level. The molecular pathway involved is only partially understood, and comprises voltage-dependent movement of serotonin and subsequent serotonergic signaling^25^. This pathway is an important proof-of-principle of activating a melanoma-like invasive phenotype in the absence of oncogenic mutation or carcinogens^26^.

Although the background rate in control populations is very low (1% of the animals), the incidence of conversion from a depolarization treatment (such as the chloride channel opener ivermectin, which in *Xenopus* depolarizes cells bearing the GlyR channel^20^,^27^) is ~98%^21^. Other treatments (e.g., those interfering with serotonergic steps that mediate bioelectric control of melanocytes) result in intermediate penetrance of the phenotype, with each of them occurring in various characteristic percentages of a treated population^21^. However, a most striking aspect is that it is all-or-none at the level of the individual. While the decision to convert or not is stochastic within the treated cohort^21^, it is always made at the level of the whole animal: no partially converted tadpoles have been seen in any of the experiments (including dozens of different reagents and means of altering serotonergic or bioelectric properties of cells *in vivo*). Each animal is either fully covered with the ectopic, highly-arborized melanocytes, or it looks completely normal (Fig. 1). The cellular network either reaches a fully-converted outcome or not, but the entire larva does it as a coordinated unit. For any given treatment, each individual makes a stochastic decision to convert or not (so that the population converts at a rate specific for that perturbation), but that decision is coordinated among all the cells. A salt-and-pepper pattern, which would be expected from cells that made independent stochastic decisions, has not been reported in this context. Despite the detailed molecular and cell-level dissection of the signaling pathway involved, the stochastic nature and whole-body coordination in this system remains largely mysterious. We thus turned to Machine Learning as a way to discover models that could help understand the global systems properties of this event.

Attempting to understand this synchronized stochasticity, a recent computational approach inferred a fully specified signaling network that quantitatively explained the dynamics of this system^21^. Using incidence of conversion from each perturbation experiment as input, together with a basic pathway that had been uncovered between the relevant signaling components^20^,^25^, the method reverse-engineered a complete signaling network including all the necessary and sufficient regulatory elements, interactions, and parameters that could recapitulate the frequencies of converted vs. wild-type larvae observed *in vivo* in each experiment.

The use of machine learning to understand complex dynamics from molecular functional data is an important strategy for addressing the gap between increasingly available genetic data and the control of large-scale system behaviors in biomedicine^28^,^29^,^30^,^31^. The ultimate goal for such systems is not only to explain available data, but to be used to rapidly identify treatments that would give rise to desired outcomes, especially in situations too complex for human scientists to infer by hand what course of action will lead to a desired systems-level response. Thus, we sought to validate the identified network and approach using a most stringent test: interrogating the network to discover a treatment that produced a capability not reached in any of the previous experiments in this field. Specifically, we asked what experimental condition could break the synchrony and achieve partially-converted tadpoles. Our results validate the approach, identifying a treatment that for the first time produced partial melanocyte conversion within individual animals.

## Results

### *In silico* discovery of a novel phenotype

**Figure 1:**
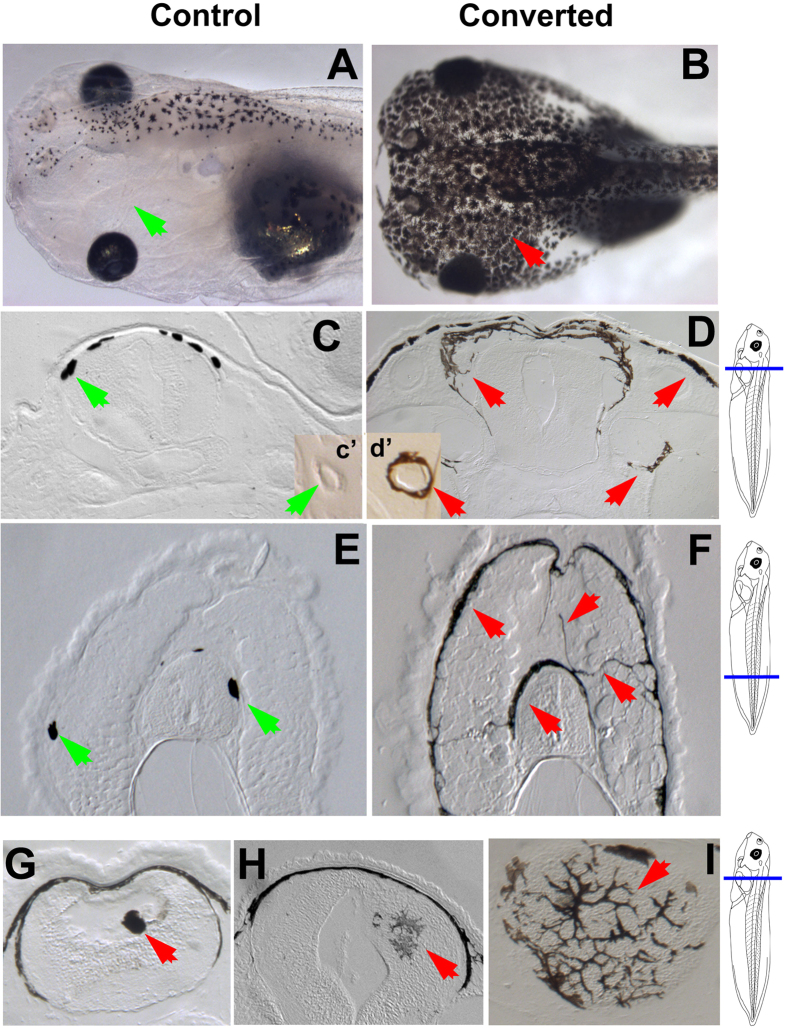
Conversion of melanocytes to a metastatic-like state. (**A**) Dorsal view of a wild-type st. 45 *Xenopus laevis* tadpole; note the small melanocytes, absent from large areas of the head (green arrowhead). (**B**) In contrast, animals resulting from any of several treatments^20^,^39^ that depolarize instructor (GlyR-expressing) cells or perturb their downstream serotonergic signaling exhibit extensive overabundance and uniform coverage with highly arborized melanocytes (red arrowhead). This occurs in an all-or-none manner in some percentage of the animals (frequency depending on the specific manipulation)^21^,^25^. Sectioning (level of section shown in schematics to the right) reveals the main features of melanoma-like phenotype: over-proliferation, arborization, and invasiveness. (**C)** Normally round melanocytes dorsal to the neural tube (green arrow) become highly arborized and drop down over the neural tube itself (**D**, red arrows). Inset panels show blood vessels, normal in c’ and covered by invasive melanocytes in d’, as occurs in melanoma. Sections further along the tail likewise show small numbers of round melanocytes in control larvae (**E**, green arrows) compared to the excess of long, abnormally extended and ectopically localized melanocytes in converted animals (**F**, red arrows). In converted animals, cells can be seen invading the lumen (**G**) or neural tissue (**H**) of the neural tube, also often forming networks (**I**) as has been observed in vasculogenic mimicry of mammalian melanoma^40^. The authors thank Vaibhav Pai for allowing us to use his drawing of a st. 45 *Xenopus laevis* tadpole in this figure.

**Figure 2:**
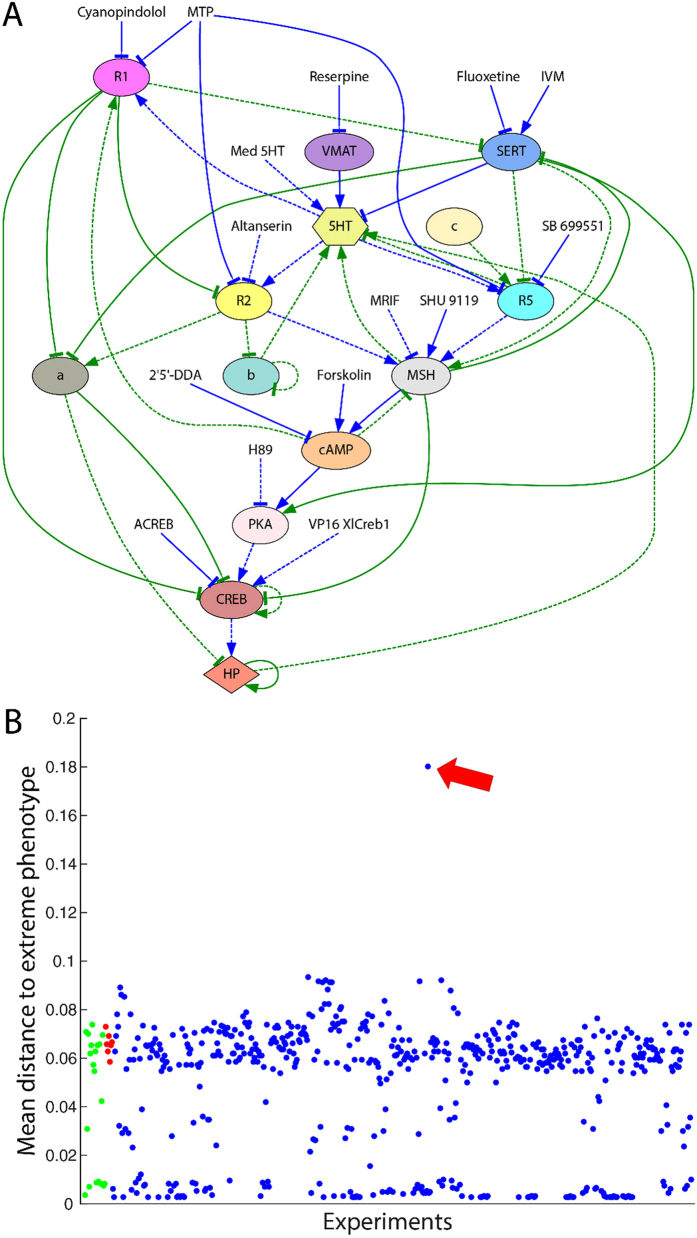
Inferred signaling network model and *in silico* predictions. (**A**) A computational method reverse-engineered a signaling network able to recapitulate the level of conversion stochasticity of a series of pharmacological experiments. (**B**) Phenotypic predictions of the model for all experimental combinations of up to three reagents. Each point represents the mean distance of 100 simulations of a specific experiment (i.e., a specific combination of reagents) to the two extreme tadpole phenotypes (normal and total conversion). Training dataset in green, validation dataset in red, new experiments in blue. Only one combination of three reagents (red arrow) is far from the two extreme phenotypes, indicating a partially converted phenotype.

We employed a stochastic model of the regulation of melanocyte conversion in *Xenopus*, uncovered by a Machine Learning approach^21^, to computationally discover a novel phenotype and the pharmacological perturbations necessary to produce it. Figure 2A shows the inferred dynamic model, including the entities used to perturb the system in past work^21^, the signaling elements participating in the pathway^20^,^25^, the incidence of conversion produced by various phenotypes, unknown required components inferred (predicted) by the system, and all the interactions between all the components. The perturbation experiments consist in the application of a precise combination of reagents during *Xenopus* development, which stochastically results in a wild-type phenotype (correct numbers of round melanocytes in their normal locations) or a completely converted phenotype (embryo covered extensively with highly-arborized melanocytes that invade many internal tissues and blood vessels) (Fig. 1). Interestingly, no intermediate phenotype was observed in the training data set (20 experiments with none, one, or two reagents applied) or the validation data set (seven experiments with two or three reagents applied)–tadpoles converted or not, stochastically but always in an all-or-none manner for each animal^21^. Could this coordination be broken, and if so, how?

We then performed *in silico* all possible experiments involving the application of up to three reagents, for a total of 576 different experiments, each repeated 100 times. Figure 2B shows, for each experiment, the average distance from the resultant phenotype (degree of conversion) to either of the extreme phenotypes: normal or complete conversion (see methods section for the exact formulation of the distance metric). In green are those experiments (combination of reagents) used in the training dataset to infer the model, in red are those used in the validation dataset, and in blue are all the experiments that had never been performed *in vivo*. The results show that all except one experiment (red arrow) are below the 0.1 threshold to an extreme phenotype (normal or converted). The experiment above the 0.1 threshold indicates an intermediate phenotype between the previously observed wild-type normal pigmentation and the converted phenotype (i.e., a novel situation in which only some melanocytes within a single animal convert to the metastatic-like phenotype). This phenotype is predicted by the inferred model to result from a combined application of altanserin (an R2 inhibitor), reserpine (a VMAT inhibitor), and VP16-XlCreb1 (constitutively active CREB).

### Dynamics of the partially converted phenotype

**Figure 3:**
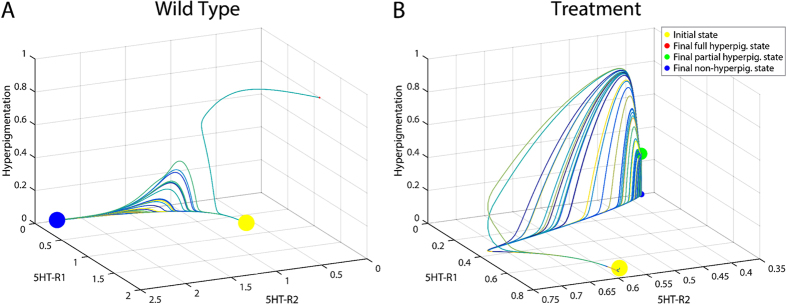
Phase space of the wild type and the experiment predicted to produce a partially pigmented phenotype. The trajectories of the state of two serotonin receptors (5HT-R1 and 5HT-R2) and the degree of conversion is shown among 100 simulations. (**A)** Without any treatment (wild type), the dynamical system transitions from the initial instable state (embryo, yellow dot) stochastically towards a final stable state with a normal conversion level (0, blue dot) or towards a final state with a high conversion level (0.92, red dot). (**B)** Applying the treatment combining the discovered three reagents (altanserin, reserpine, and constitutively active CREB), the dynamics reveal a bifurcation in the dynamical system and the appearance of a new attractor with a partial conversion level (0.25, green dot) in addition to another attractor with a normal conversion level (0, blue dot). The attractor dot size is proportional to the number of converging trajectories to that final state.

We first investigated *in silico* the dynamic characteristics of the predicted partially converted phenotype with the inferred model using the combined application of the drugs altanserin and reserpine, and constitutively active CREB. Figure 3 shows the phase portraits of 100 simulations of the wild type and the treated embryos with the trajectories of the state of two serotonin receptors (5HT-R1 and 5HT-R2) and the degree of conversion through time. The simulation starts in the instable initial state (yellow dot) and, due to the inherent stochasticity of the model, ends in one of several stable attractors, representing a final partial converted state. Without treatment (wild type, Fig. 3A), the embryos stochastically develop normally (blue dot, 99% of simulations) or high conversion levels (red dot, 1% of simulations). In contrast, applying the discovered treatment with three drugs, the embryos stochastically develop with an intermediate conversion level (green dot, 75% of simulations) or a normal conversion level like the wild type (blue dot, 25% of simulations).

The “partially converted” attractor (green dot) was not observed in any previous simulation or experiment *in vivo*^21^, representing a novel bifurcation in the signaling network’s dynamics due to the specific perturbation caused by the application of the three reagents. In this way, the inferred model predicted a partially converted phenotype at about 25% between wild-type and totally converted.

**Figure 4:**
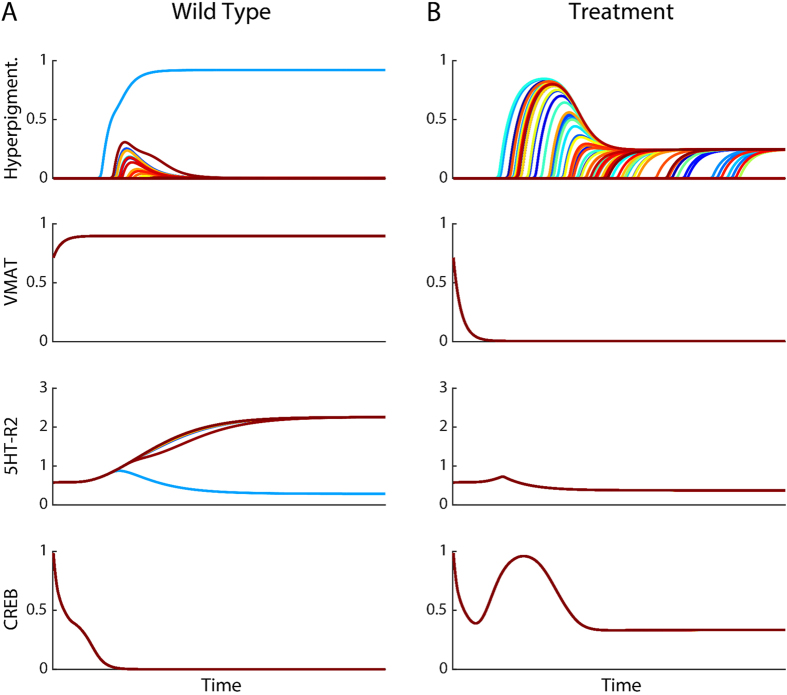
Dynamics of the degree of conversion and perturbed pathway components in the untreated and treated experiments. The panels show the dynamics of the degree of conversion and levels of the affected components by the drugs in the treatment through time over 100 simulations. (**A)** With no treatment (wild type), only extreme phenotypes (normal or high conversion levels) are stochastically produced. VMAT converges to high levels, R2 to stochastically either high or low levels, and CREB to low levels. (**B)** The treatment combining reserpine (inhibiting VMAT), altanserin (inhibiting R2), and constitutively active CREB (increasing CREB) produces the expected changes in the levels of the affected pathway components, lowering the levels in the case of inhibitors (VMAT and R2) or increasing them in the case of adding constitutively active CREB. Notice that in both A and B there are trajectories converging at 0 conversion level (normal).

The effects of the three reagents in the treatment can be observed in the temporal dynamics of the level of conversion and pathway components targeted by the drugs (Fig. 4). Embryos with no treatment (wild type, Fig. 4A) stochastically develop normal levels of conversion (99% of the simulations) or high levels of conversion (1% of the simulations). These embryos always have high levels of VMAT and low levels of CREB. However, the levels of serotonin receptor R2 are stochastically either low (blue line, corresponding to high levels of conversion) or high (all other colors, corresponding to normal levels of conversion). Embryos under the three drugs treatment (Fig. 4B), stochastically develop either the normal level of conversion (25% of the simulations) or the partial conversion phenotype (75% of simulations). The plots show how the inhibition drugs reserpine and altanserin causes VMAT and R2, respectively, to converge to a low level, whereas the constitutively active CREB causes CREB levels to reach a high transient state and then converge to a lower level, but overall staying higher than the wild type.

### *In vivo* validation of the computationally discovered treatment and phenotype

**Figure 5:**
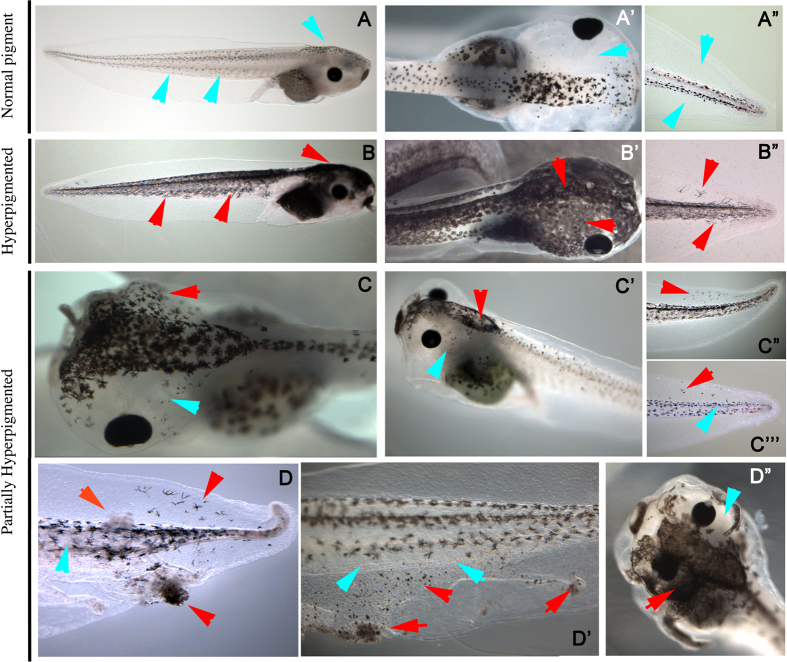
*In vivo* validation of the computationally discovered partially converted phenotype. Normal melanocytes are indicated with blue arrowheads, while areas populated with converted melanocytes are indicated with red arrowheads. (**A)** Control animals exhibit small, round melanocytes confined to stripes around the spinal cord and over the brain (**A**). Note the absence of melanocytes in the periocular region (**A**’). In normal animals’ tails, melanocytes never exit the stripes of axial muscle (do not venture into the fin). (**B)** Converted animals produced by Ivermectin-induced depolarization exhibit spread-out (arborized) melanocytes all along the flank (**B**) and around the eyes (**B**’)–they colonize every region of the head. The arborized melanocytes also leave the mid-body and colonize the fin (**B**”). (**C)** In contrast, embryos treated with VP16-XlCreb1and exposed to 5HT-R2 and VMAT blockers revealed a never before-seen phenotype where some regions of the animal were converted (**C**, periocular region of the right eye, red arrowhead), and some were wild-type (**C**, periocular region of left eye, blue arrowhead). This can be also seen in the transformation of cell shape over the brain (**C’**, red arrowhead) but normal melanocyte morphology elsewhere (**C**’, blue arrowhead). In the tail (**C**”,**C**’”) we observed some cells invading the fin, but they had normal morphology. We observed a great diversity of different combinations that were intermediate between normal and converted outcomes (only a representative sample is shown here), which otherwise never occurred in the same animal. (**D)** Furthermore, we noticed a new, cancer-like behavior that had not occurred in any previous experiment. This included the induction of individual nodules, which were both pigmented and unpigmented (**D,D**’); this occurred in ~10% of the animals that likewise exhibited partial conversion (**D**”, red arrowhead region vs. blue arrowhead region).

We then sought to validate the partially converted phenotype predicted by the inferred model by applying *in vivo* the newly discovered combination of reagents. Control embryos exposed to vehicle alone were 99% normal (N = 258, Fig. 5A-A”). Embryos exposed to ivermectin (which depolarizes instructor cells; the effect has been characterized extensively^20^,^25^) exhibited 97% conversion (N = 313, Fig. 5B-B”). In contrast, embryos expressing constitutively active CREB (tom-VP16-XlCreb1) and simultaneously treated with a cocktail of Altanserin (R2 blocker, 10 uM) and Reserpine (VMAT blocker, 100 uM) exhibited the predicted never before-seen partial conversion in 22% of the animals (N = 167, Fig. 5C-D”), while 24% were completely normal, and 54% exhibited the standard full conversion phenotype.

## Discussion

Here, we have presented the discovery of a novel *in vivo* phenotype, and the precise intervention required to obtain it, by means of a computational methodology. From a reverse-engineered signaling network able to recapitulate the stochasticity of two phenotypes representing wild-type and extreme levels of melanocyte conversion in *Xenopus laevis* larvae, we sought to discover a pharmacological intervention that could generate a never-seen partially converted phenotype. We performed *in silico* all the possible experiments combining one, two, or three reagents and found that only one specific combination that was able to produce an intermediate phenotype. The model dynamics of this intervention showed that this precise combination produced a bifurcation in the dynamical system, generating an attractor corresponding to a phenotype 25% in between the known wild type and totally converted phenotypes. Almost a decade of highly diverse experiments focused on this pathway had not revealed such an outcome^20^,^25^. While detailed mechanistic dissection of the pathway explained signaling on a single cell level, explanations for the stochastic concordance throughout each animal, the possibility of partial conversion, or the reagents that could achieve it, had all remained unknown without the described automated approach that strongly augmented human analysis of this complex dynamical system.

We validated these predictions *in vivo*, confirming the *in silico* discovery of the novel phenotype. When we applied the discovered combination of three reagents, two drugs and constituently active CREB, we observed for the first time the predicted partial converted phenotype–animals in which some melanocytes and melanocyte-free regions were normal, while others were converted and colonizing ectopic sites. It is crucial to note that each of these reagents alone causes some incidence of full conversion–the individual reagents have already been validated to work in *Xenopus* (i.e., they do penetrate the embryo, reach target cells, and affect this pathway sufficiently to fully convert some percentage of each treated sample)^21^; however it is of an all-or-none character. Moreover, we have previously shown that injection of just a few cells in the embryo (for example, with depolarizing reagents) is sufficient to cause complete conversion^21^,^25^–the partial conversion here is *not* due to mosaic expression of the CREB construct, as injected alone, in one cell of the 32-cell stage embryos as above, causes an incidence of ~45% *complete* conversion. The model predicted the wild-type phenotype within 1% (24% *in vivo* vs 25% *in silico*), yet it aggregated the partially and fully converted phenotypes observed *in vivo* (22% partial, 54% fully converted) into a single partial phenotype (75% partial). This difference in the frequency of partial and fully converted phenotypes between the *in silico* and *in vivo* results may be explained by the lack of time information in the training dataset and the observed dynamics of the system (Figs 3 and 4). In particular, a number of trajectories ending in the partially converted phenotype actually are crossing the levels of highly converted phenotypes. This observation may indicate that the full conversion that we observed *in vivo* may be transient. Further investigations, and methods that enabled the tadpoles to survive to older stages for tracking any subsequent changes in melanocyte status, would be necessary to validate predictions of transient (time-dependent) changes of the phenotypes predicted by the model. Indeed, the training dataset does not contain any time information, and hence the current system does not yet capture realistic timing, which would allow a finer-course analysis of trajectories and comparison with *in vivo* results; in future work we will extend this platform to include time-series data, so that these transient phenotypes can be better investigated.

Identification of interventions that alter system-level physiology or pattern formation is a crucial nascent field with many implications for biomedicine^32^,^33^. The complexity of biological regulation makes it very hard to completely and mechanistically understand signaling networks, the prediction of resultant experimental outcomes, and hence the discovery of novel phenotypes. Computational dynamic modeling of complex regulatory networks is essential for the discovery of new capabilities of biological systems and prediction of specific perturbations that result in desired outcomes. Using these models, we can computationally and systematically interrogate the system to find the precise interventions necessary for the development of specific phenotypes^33^. The computational methodology presented here shows how a reverse-engineered signaling system can be used in this way to precisely find a set of reagents that produce a never before seen outcome at the intersection of embryogenesis and metastatic-like conversion. These computational methods are paving the way for the discovery and further understanding and manipulations of multicellular, stochastic, and multidimensional biological phenomena, as well as for the discovery of novel and effective therapies that target cooperative or group aspects of cell function *in vivo*.

## Methods

### Animal husbandry

*Xenopus* embryos were maintained according to standard protocols^34^ in 0.1× Marc’s Modified Ringers (MMR; pH 7.8). *Xenopus* embryos were staged according to Nieuwkoop and Faber^35^. All experimental procedures involving *Xenopus* embryos were approved by the Institutional Animal Care and Use Committees (IACUC) and Tufts University Department of Laboratory Animal Medicine under protocol M2014-79. All experiments were performed in accordance with relevant guidelines and regulations. Although we have not observed light to make any difference to the converted phenotype, all groups were subjected to the same light conditions.

### Microinjection, drug exposure, sectioning, and image collection

Embryos were injected with mRNA encoding constitutively active CREB (tom-VP16-XlCreb1^36^) at 1/32 cell stage and then exposed to a mix of Altanserin (5HT-R2 blocker, 10 uM) and Reserpine (VMAT blocker, 100 uM) using the same methods described previously^20^,^21^,^37^,^38^. Sections were made at 40 μ on a Leica vibratome after JB4 embedding. Images were collected using a Nikon SMZ-1500 microscope and each phenotype was visually characterized as having normal melanocyte status, partial conversion, or full conversion; these three classes are discrete and readily distinguished as categorical data; the quantification of the definition of these states was described in refs 20 and 39.

### Dynamic signaling model of melanocyte conversion

We used the dynamic signaling model of *Xenopus* melanocyte conversion presented in ref. 21. The model is based on Hill-kinetics and includes 14 stochastic ordinary differential equations describing the interactions of signaling molecules, pharmacological compounds (drugs, set to 0 when not applied and 1 when applied), and the level of melanocyte conversion (the phenotype, equivalent to the pigmentation level) of a developing embryo. The interaction between signaling molecules and drugs are modeled with a combination of Hill functions, each of them representing the interaction between two products. When multiple interactions regulate a product, they can be grouped as sufficient, necessary, or a combination of them. Sufficient interactions can regulate the production of the target product independently of other sufficient interactions (modeled with a max operator, similar to a logic OR interaction; hence, activation has preference over inhibition). In contrast, all the necessary interactions need to be coordinated to regulate the production of the target product (modeled with a min operator, similar to a logic AND interaction; hence, inhibition has preference over activation). This scheme allows the modeling of a rich variety of regulatory interaction combinations. In addition, each product includes a decay term (with a specific decay constant) and a Gaussian random noise of zero mean to model the stochasticity of the system.

The following set of differential equations, including all the kinetic parameters, define the complete model (*ξ(t*) represents a Gaussian random noise of zero mean):


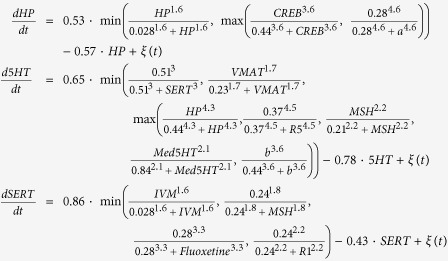



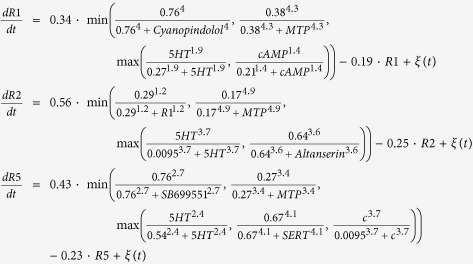



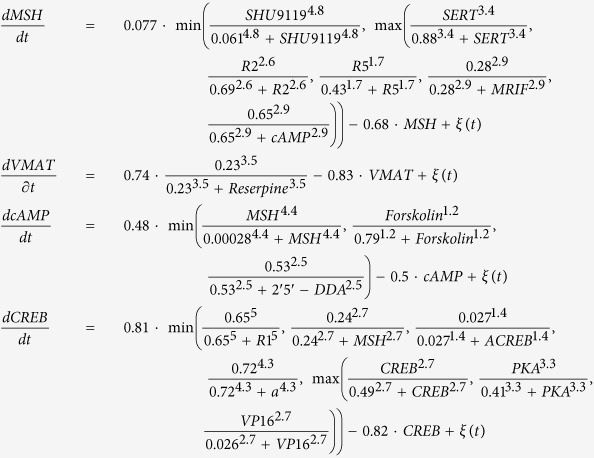



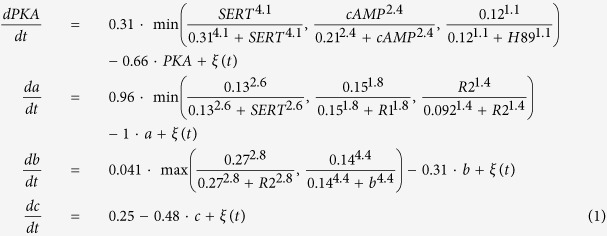


This set of equations (defined in the model) together with a set of initial conditions of the signaling products (defined in the model) and the drugs (defined in the experiment) form an initial value problem which is then numerically solved until reaching a steady state. Due to the stochastic nature of the equations, different runs with the same initial conditions (same experiment) can result in different phenotypic outcomes (level of melanocyte conversion) as observed *in vivo*. The model was reverse-engineered directly from a dataset of 20 experiments combining different pharmacological drugs and resulting in a specific frequency of two different and extreme phenotypes (within each phenotype, the animal-to-animal variation in pigmentation level was not visually detectable): either normal (pigmentation level 0) or high level (pigmentation level 1) of melanocyte conversion, never an intermediate level as described in ref. 21. The model was further validated with an additional dataset of 7 experiments not used during the training^21^. The model kinetic constants, also inferred from the experimental data, are in arbitrary units.

### Phenotype distance metric

To discover phenotypes different from the two extreme levels of conversion (normal or high) previously observed *in silico* with the model and *in vivo* with *Xenopus* experiments, we defined a distance metric for simulated phenotypes obtained with the model. The metric quantifies the level of intermediate conversion obtained in a set of resultant *in silico* phenotypes simulated with the model for a specific experiment (combination of reagents). The distance metric is defined with the following equation:


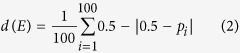


where *d(E*) is the mean distance of experiment *E* (a combination of reagents) when simulated 100 times with the model and resulting in phenotypes with level of conversion *p*_*i*_ (the level of conversion is a dynamic variable in the model, with values from 0 for the wild type to 1 for a total conversion).

## Additional Information

**How to cite this article:** Lobo, D. *et al*. Discovering novel phenotypes with automatically inferred dynamic models: a partial melanocyte conversion in *Xenopus. Sci. Rep.* **7**, 41339; doi: 10.1038/srep41339 (2017).

**Publisher's note:** Springer Nature remains neutral with regard to jurisdictional claims in published maps and institutional affiliations.
